# Transcriptomic Analysis of the Underground Renewal Buds during Dormancy Transition and Release in ‘Hangbaishao’ Peony (*Paeonia lactiflora*)

**DOI:** 10.1371/journal.pone.0119118

**Published:** 2015-03-19

**Authors:** Jiaping Zhang, Yun Wu, Danqing Li, Guanqun Wang, Xin Li, Yiping Xia

**Affiliations:** 1 Institute of Landscape Architecture, College of Agriculture and Biotechnology, Zhejiang University, Hangzhou, Zhejiang, People’s Republic of China; 2 Department of Biochemistry and Molecular Biology, College of Life Sciences, Nanjing Agricultural University, Nanjing, Jiangsu, People’s Republic of China; University of Western Sydney, AUSTRALIA

## Abstract

*Paeonia lactiflora* is one of the most famous species of herbaceous peonies with gorgeous flowers. Bud dormancy is a crucial developmental process that allows *P*. *lactiflora* to survive unfavorable environmental conditions. However, little information is available on the molecular mechanism of the bud dormancy in *P*. *lactiflora*. We performed de novo transcriptome sequencing using the Illumina RNA sequencing platform for the underground renewal buds of *P*. *lactiflora* ‘Hangbaishao’ to study the molecular mechanism underlying its bud dormancy transition (the period from endodormancy to ecodormancy) and release (the period from ecodormancy to bud elongation and sprouting). Approximately 300 million high-quality clean reads were generated and assembled into 207,827 (mean length = 828 bp) and 51,481 (mean length = 1250 bp) unigenes using two assembly methods named “Trinity” and “Trinity+PRICE”, respectively. Based on the data obtained by the latter method, 32,316 unigenes were annotated by BLAST against various databases. Approximately 1,251 putative transcription factors were obtained, of which the largest number of unique transcripts belonged to the basic helix-loop-helix protein (bHLH) transcription factor family, and five of the top ten highly expressed transcripts were annotated as dehydrin (DHN). A total of 17,705 simple sequence repeat (SSR) motifs distributed in 13,797 sequences were obtained. The budbreak morphology, levels of indole-3-acetic acid (IAA) and abscisic acid (ABA), and activities of guaiacol peroxidase (POD) and catalase (CAT) were observed. The expression of 20 interested unigenes, which annotated as DHN, heat shock protein (HSP), histone, late elongated hypocotyl (LHY), and phytochrome (PHY), and so on, were also analyzed. These studies were based on morphological, physiological, biochemical, and molecular levels and provide comprehensive insight into the mechanism of dormancy transition and release in *P*. *lactiflora*. Transcriptome dataset can be highly valuable for future investigation on gene expression networks in *P*. *lactiflora* as well as research on dormancy in other non-model perennial horticultural crops of commercial significance.

## Introduction

Herbaceous peonies belong to the genus *Paeonia*, which is the sole genus in the family Paeoniaceae[1]. The species *Paeonia lactiflora*, which is well known for its highly medicinal value and attractive flowers, is mainly distributed in temperate areas worldwide, and has been cultivated as a medicinal and ornamental plant[2,3] in China for nearly 4,000 years[4] (2,000 years reported in some studies)[5,6]. Herbaceous peonies are geophytes and perennials, as such, their aboveground organs dry completely and underground crowns (tuberous compressed underground rhizome) enter dormancy in the autumn[7]. A number of underground renewal buds, generated on the crown surface[8], differentiate into primordia of vegetative and reproductive organs. When the day length and temperature decrease between August and October in autumn, the buds appear to enter a stage of endodormancy, but eventually shift to a stage of ecodormancy under cold stress in December and January for over-wintering in China. Most large buds develop into aboveground parts again in the spring with rise in temperature[1,8–10].

A certain amount of chilling is crucial for the bud dormancy transition (the period from endodormancy to ecodormancy) and release (the period from ecodormancy to bud elongation and sprouting) of *P*. *lactiflora*[1,7,10]. The wild populations and most cultivars of *P*. *lactiflora* are mainly distributed or planted in the north, northwest, northeast and southwest of China, most of which are cool- or temperate-climate regions[7,9]. Muggy climate seriously hinders its cultivation and application in the south Yangtze River regions, such as the Zhejiang Province. Furthermore, winters are frequently warm because of global warming trends, and delayed autumn and advanced springtime occur every year, which causes shortage of chilling accumulation[11]. Chilling deficit results in uneven bud breakage, abnormal leaves and few flowers in *P*. *lactiflora*. These defects greatly influence the ornamental value of *P*. *lactiflora* used as cut-, potted- and garden-flower, and limit its sustainable production and landscape utilization[12–14]. Therefore, optimized cultivation strategies for effective control of dormancy, and breeding of cultivars with low chilling requirements are urgently needed in Zhejiang Province and other regions in the south of Yangtze River in China. Comprehensive studies on molecular mechanisms of *P*. *lactiflora* underlying its bud dormancy are prerequisites for achieving these goals.

During the last two decades, many studies on gene expression and transcriptional response to low temperature and their regulation have been carried out in the model plant *Arabidopsis*[15–17]. Among horticultural crops, studies on the mechanism of dormancy transition and release have been conducted in fruit trees such as *Pyrus pyrifolia*[11,12], *Prunus mume*[13,18] and *Vitis riparia* [14], etc. Research on dormancy mechanism has also focused on some economic and ornamental geophytes, such as *Allium wakegi*[19], *Allium sativum*[20,21], *Solanum tuberosum*[22] and *Tulipa gesneriana*[23]. Within the genus *Paeonia*, much progress has also been made in understanding the morphological[24,25], physiological[26] and molecular aspects[27,28] of tree peonies. Gai et al. (2011 and 2013) investigated the transcriptome and transcript profiles of flower buds of *Paeonia ostii* during dormancy release induced by artificial chilling[29,30]. For herbaceous peonies, studies on their chilling requirements have been conducted mainly based on morphological observations[1,3,5,7,9,10,31,32]. However, the relative dearth of molecular studies has occluded a comprehensive understanding of dormancy transition and release mechanism in herbaceous peonies.

**Fig 1:**
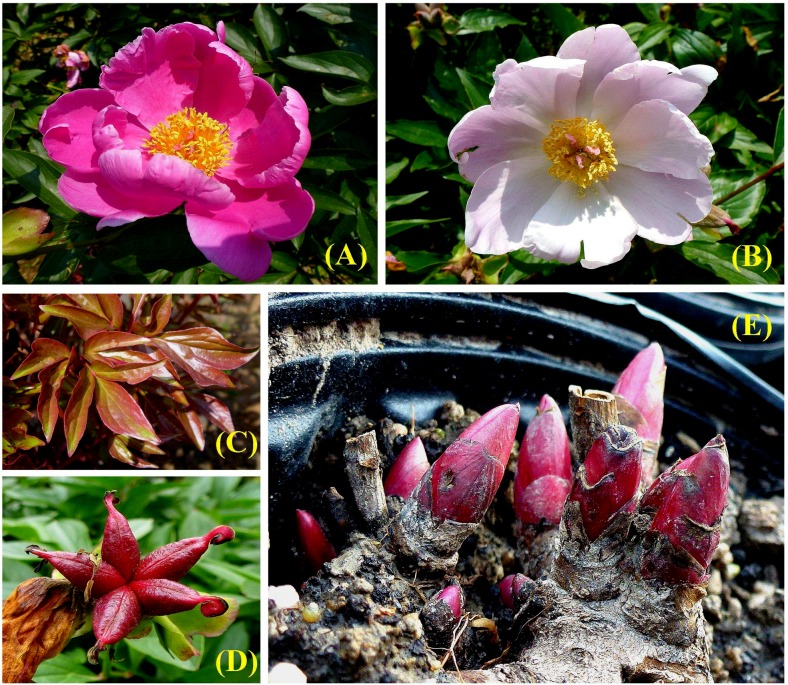
Ornamental organs and experimental samples of *P*. *lactiflora* ‘Hangbaishao’. **(A)** The pink purple flower in the early florescence stage. **(B)** The pink white flower in the end of florescence stage. **(C)** The purple red young leaves. **(D)** The purple red ripe fruits. **(E)** The underground renewal buds sampled for deep sequencing and transcriptome research.

In this study, we selected a local cultivar of *P*. *lactiflora*, called ‘Hangbaishao’ in Chinese by the people in Zhejiang, as the experimental material[33]. Although cultivation of herbaceous peonies is rather rare in Zhejiang, only *P*. *lactiflora* ‘Hangbaishao’ has adapted to the muggy climate and grown vigorously for many years in this province. According to our previous observation, ‘Hangbaishao’ could sprout, grow and bloom quite well compared with many other ornamental cultivars of *P*. *lactiflora* under the relatively high winter temperature of Hangzhou (the provincial capital of Zhejiang Province). It has good ornamental value with flowers (simple form, over 10 petals with variable colors which are pink purple in the early florescence stage, pink in the full and pink white in the end) (Fig. 1A and Fig. 1B), young leaves (Fig. 1C) and even ripe fruits (Fig. 1D). The steamed and dried root of ‘Hangbaishao’ and some other medicinal cultivars of *P*. *lactiflora* is white, so the root is often referred to as “Bai-shao”[34], “Radix Paeoniae Alba”[34] or “White peony root”[35] in some literatures, and has always been used as herbal supplement. Therefore, ‘Hangbaishao’ is an ideal and valuable material with strong local adaptability, relatively low chilling requirements and multiple uses to study the dormancy mechanism of herbaceous peonies in the winter in warm temperate or subtropical areas, such as Zhejiang Province. Furthermore, it can potentially be used for breeding new cultivars with high ornamental and medicinal values, low chilling requirements and strong heat resistance.

Our study of the bud dormancy mechanism for ‘Hangbaishao’ consisted of four sets of experiments: first, morphological observation for three to four years of bud sprouting, shoot elongation and flowering under natural or artificial chilling and different growth regulator treatments, and study of the chilling requirements for dormancy release and budbreak; second, physiological measurements of buds under different treatments; third, transcriptome sequencing of dormant buds by RNA-Seq[36]; and fourth, transcript profiling and functional mining of genes closely related to bud dormancy. In this paper, we mainly present our findings on the transcriptome sequencing of ‘Hangbaishao’ bud dormancy transition and release. We sequenced cDNA libraries from the underground renewal buds in six dormant phases using Illumina deep-sequencing technology, obtained nearly 300 million clean reads, and submitted the sequences to the National Center for Biotechnology Information (NCBI). This is the first study on dormancy based on transcriptome level for herbaceous peonies of the Paeoniaceae family.

## Materials and Methods

### Plant material

Pan’an County (E 120°17’-120°47’, N 28°49’-29°19’), which is in the middle of Zhejiang Province[33], is the major *P*. *lactiflora* ‘Hangbaishao’ producing area of China, and is reputed to be “The hometown of Chinese medicinal materials”. In the year 2012, divided crowns of three- or four-year-old ‘Hangbaishao’ were introduced from Pan’an to Perennial Flower Resources Garden of Zhejiang University in Hangzhou (E 118°21’ -120°30’, N 29°11’ -30°33’). All crowns were planted outdoors in one gallon pots (one pot per crown). The culture medium consisted of 7 garden soil: 2 peat: 1 perlite (by volume). The crowns were cultivated under natural light and normal watering without chemical treatment. The natural temperature was recorded once per hour by the ZDR-20 temperature-humidity recorder (Hangzhou Zeda Instruments Limited Company, Hangzhou, China).

### Treatment and morphological observation

The dormant crowns of potted ‘Hangbaishao’ were transferred into a glasshouse (held at 15–25℃, nature light levels, watering twice or three times a week) at 2 or 3 week intervals from November 26, 2012 to March 11, 2013. The specific transfer-dates were November 26, December 10, December 24 in the year 2012, and January 7, January 21, February 4, February 25, and March 11 in 2013, respectively. We defined these eight natural chilling treatments as Treatment 1 to 8 (Table 1, Tre. 1 to 8 for short). There were three replicates (7 pots/replicate) in each treatment group.

Four morphological indices were observed to evaluate the phase of dormancy release: Bud Break Percentage checked five weeks after being transferred into glasshouse (BBP); average Number of Sprouted Buds per plant (NSB); Days number between the transfer-date and date of the First stem Elongated visibly (DFE); and Days number between the transfer-date and date of stem Elongated visibly of All plants (DEA)[3,5,7,9,32,37]. The beginning of budbreak was defined as bud scales opened and tender shoots or leaves emerged[9,10,12,30,37]. If a plant in a pot grew one (or more) sprouted bud(s), we defined it as a sprouted plant. The experiment was performed in a completely randomized design. Analysis of variance (ANOVA) was used to determine the statistical significance of differences in indices.

### Sample collection and selection

As mentioned before, the pots were transferred into glasshouses eight times from November 26, 2012 to March 11, 2013. We collected the underground buds at each transferring date as materials for transcriptomic and physiological studies. There were only two to five large buds on the surface of crowns, so bud collection may negatively influence the subsequent observation and measurement steps. Therefore, in each treatment, another 20 pots of dormant crown with the identical treatment were assigned specifically for sample collection. The underground buds of these 20 crowns were sampled when the pots were transferred into glasshous. Two to four full and robust buds for each crown were collected (Fig. 1E, vertical diameter × transverse diameter ≥ 1.5 cm × 0.5 cm[38–40]). After quick-freezing in liquid nitrogen, the samples were stored at—80°C until RNA extraction. These buds were also used for subsequent physiological and biochemical experiments.

Sample selection for RNA extraction and transcriptome sequencing was based on morphological observation. Considering that Tre. 4 was the intermediate status beween Tre. 3 and Tre. 5, and because the morphological differences among them were not significant (data shown in Results and Discussion), we did not use the sample from Tre. 4 for transcriptome research. We also did not use the sample from Tre. 8 for similar reasons. We chose the buds from six other treatmens (Tre. 1 to 3, and Tre. 5 to 7) for RNA extraction and transcriptome sequencing. However, on the other hand, we used the buds from all eight treatment conditions in physiological or biochemical experiments that will be mentioned later.

### RNA extraction, cDNA library construction and deep sequencing

The underground buds sampled from six sampling dates in six treatments were assigned to six independent pools. Total RNA was extracted from approximately 0.5 g of frozen sample using TRIzol reagent (Invitrogen, Carlsbad, CA, USA), with a phenol/chloroform/isoamyl alcohol (49:49:2) pretreatment extraction step. RNA was purified using an RNeasy Mini Kit (Qiagen, Valencia, CA, USA), and quality-checked using an Agilent 2100 Bioanalyzer (Agilent Technologies, Santa Clara, CA, USA). Good samples with RNA integrity number ≥ 7, 28S:18S > 1, OD260/280 ≥ 2 and OD260/230 ≥ 2 were utilized, and mixed in equal proportions to construct a normalized cDNA library.

The poly(A)-containing mRNA was purified using poly-T oligo-attached magnetic beads, and then fragmented into short fragments. About 180 bp fragments were used as templates to synthesize first-strand cDNA with random hexamer primers. Second-strand cDNA synthesis was performed using DNA polymerase I, dNTPs, and RNase H. The products were amplified by PCR and purified after end repair and adaptor ligations by an Illumina TruSeq RNA Sample Preparation Kit (Illumina Inc., San Diego, CA, USA). The cDNA libraries were sequenced on a HiSeq 2000 Sequencing platform (Illumina Inc., San Diego, CA, USA) using a 100 bp paired-end approach.

All of our clean sequencing data and assembled unigenes have been submitted to the Sequence Read Archive (SRA) and Transcriptome Shotgun Assembly (TSA) Database at NCBI, respectively, and are available under the BioProject accession number PRJNA245064. The SRA run accessions (SRR) of the clean sequencing data derived from Tre. 1, Tre. 2, Tre. 3, Tre. 5, Tre. 6 and Tre.7 are SRR1258112, SRR1258117, SRR1269644, SRR1269649, SRR1269650 and SRR1269651, respectively. The TSA project has been deposited at DDBJ/EMBL/GenBank under the accession GBFN00000000. The version described in this paper is the first version, GBFN01000000.

### *De novo* assembly

After removing all adaptor sequences, low quality reads, and empty reads (Q < 30 and length < 50 bp) with a FASTX-Toolkit, clean reads were *de novo* assembled using Trinity and CAP3 with default parameters. The TransDecoder, a plugin for Trinity, was used to predict optimal open reading frame (ORF) information with 80 AA minimum protein lengths. Given that the unigenes assembled only using Trinity were shorter and more fragmented, we also adopted another assembler named Paired-Read Iterative Contig Extension (PRICE), which was released in 2013[41], to extend and assemble contigs generated by Trinity. We used paired-read information to iteratively increase the length of existing contigs for six “cycles” with the following parameters:-fpp 180 99-mol 35-mpi 99-MPI 99-target 99 1 2 2—a 6. In each cycle of assembly, we mapped the reads to existing contigs, assembled the paired-ends of those reads and the contig itself to create a longer contig, constructed scaffolds and connected multiple seed contigs that could then be assembled together into a single sequence, avoided spurious assemblies that could be created by multicopy genetic elements, evaluated the output sequences to determine which was relevant to the original target of our assembly, and eliminated redundant output sequences. Each cycle contained these steps to extend the length of existing contigs assembled by Trinity, and subsequent cycles repeated the above procedure using the output of the prior cycle as input[41].

Thus, we used two methods (“Trinity” and “Trinity+PRICE” for short) to assemble clean reads into unigenes. All data on the ‘Hangbaishao’ transcriptome described in this paper, such as functional annotation and microsatellite mining, were generated by the latter method “Trinity+PRICE” unless otherwise specified.

### Unigene functional annotation

The merged and filtered unigene sequences were aligned by BLASTx (an E-value ≤ 1.0 e^-5^ was used as the cut-off line) to the NCBI non-redundant protein database (NR), Swiss-Prot protein database, Kyoto Encyclopedia of Genes and Genomes (KEGG), and Clusters of Orthologous Groups of proteins (COG). Interproscan v5 was used to predict the Pfam domain and superfamily. The proteins with the highest sequence similarity, and species distribution of the top Basic Local Alignment Search Tool (BLAST) hits for all homologous sequences were retrieved for analysis. Pathway assignment was performed on the KEGG Automatic Annotation Server (KAAS). The region of coding sequences (CDS) were obtained based on TransDecoder and translated into amino sequences with the standard codon table. With NR annotation, the Blast2GO program was used for gene ontology (GO) annotation of unigenes. The WEGO software was used for GO functional classification of all unigenes to understand the distribution of gene functions of species from a macro level. Some proteins could be identified as putative transcription factors (TFs) or regulators that belong to different families, and the ten most highly expressed transcripts were also summarized and analyzed. Unigenes were submitted to http://www.ncbi.nlm.nih.gov/Structure/bwrpsb/bwrpsb.cgi and the KAAS to predict possible COG functional classification and KEGG pathways.

### Microsatellite mining

The MISA scripting language (http://pgrc.ipk-gatersleben.de/misa/) was used to detect potential microsatellite repeats[42–44]. Simple Sequence Repeat (SSR) loci containing perfect repeat units of one to six nucleotides were mined. The minimum SSR length criteria were adjusted for the identification of perfect di-, tri-, tetra-, penta-, and hexanucleotide motifs with a minimum of 6, 5, 4, 4, and 4 repeats, respectively. The maximum number of bases interrupting two SSRs in a compound SSR was 100. Total size (bp) of examined sequences and total number of identified SSRs, SSR-containing sequences, sequences containing more than one SSR, and SSRs present in compound formation were identified. The types and frequencies of major repeats, including mononucleotide repeats, are listed.

### Biochemical observation

Based on the results of KEGG analysis in this study, we also focused our analyses on some pathways that may be important for the transition and breaking of bud dormancy, including carbohydrate metabolism, plant hormones and antioxidant response-related pathways. The proportions of sequences related to these pathways were obtained by BLAST. The biochemical levels of four key indices of plant hormones and antioxidant enzymes were also measured in order to study bud dormancy physiology and biochemistry: the contents of two important phytohormones, namely indole-3-acetic acid (IAA) and abscisic acid (ABA), were measured by enzyme-linked immunosorbent assay (ELISA) technique[45]; the activity changes of guaiacol peroxidase (POD) and catalase (CAT) were observed based on the methods described by Zhu et al.[46]. The samples from all eight treatments, including Tre. 4 and 8, were used. Approximately 0.5 g of the underground buds was prepared for each sample in all measurements.

### Analysis of interested differentially expressed genes

The number of differentially expressed genes (DEGs) between any two of six independent gene libraries were identified (False Discovery Rate (FDR) < 0.05), in order to estimate the different dormant stages (endodormancy, ecodormancy and bud elongation and sprouting) of ‘Hangbaishao’ underground buds in combination with morphological and biochemical data. Main physiological and biochemical metabolisms during five adjacent dormant phases were analyzed based on KEGG pathway annotation. The expression of 20 interested unigenes (most are DEGs), which annotated as dehydrin (DHN), metallothionein, D-galacturonic acid reductase 1, IAA, POD, heat shock protein (HSP), histone, late elongated hypocotyl (LHY) and phytochrome (PHY), were evaluated by the value of fragments per kilobase of exon model per million mapped reads (FPKM) value.

## Results and Discussion

### Evaluation of bud dormancy release and sample selection

**Fig 2:**
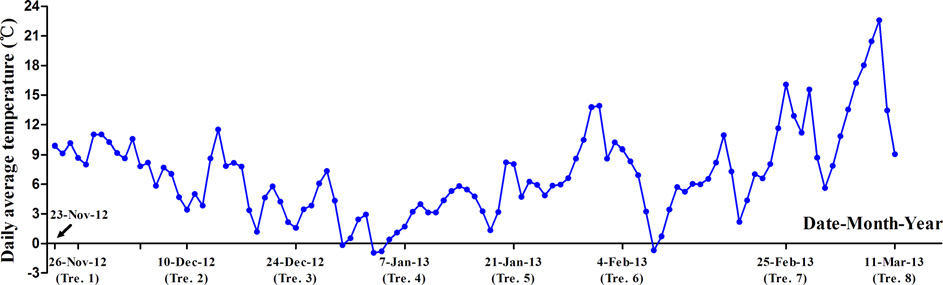
Change in the daily average temperatures (℃) in sampling base from November 23, 2012 to March 11, 2013.

We observed four critical morphological indices in order to investigate the time of bud dormancy release, and select samples with significant difference to perform transcriptome research (Table 1). The change in daily average temperature is shown in Fig. 2. Results indicated that different accumulations of natural chilling significantly influenced the percentage of ‘Hangbaishao’ budbreak and the number of sprouted buds (Table 1). The values of BBP and NSB gradually rose, whereas DFE and DEA shortened as the delay in transfer-date. The first bud of Tre. 4 sprouted and inner tender stem elongated visibly only 18 days after being transferred into glasshouse with elevated temperature. There were no significant differences in BBP, NSB and DFE after January 7, 2013 (the sampling time of Tre. 4), thus we can learn that chilling accumulation was enough for the buds of Tre. 4 to break dormancy. DEA had a sharp decline in the time of Tre. 5 compared with that of Tre. 4 (17.33 d, Table 1). That means if we need all dormant buds of ‘Hangbaishao’ to sprout more rapidly after being transferred into glasshouse, we should put them outside in the cold environment until the late January in Hangzhou City, in order to ensure they acquire more sufficient chilling accumulation.

**Table 1:** Morphological observation for dormancy release evaluation of ‘Hangbaishao’.

Treatment	Transfer-date^Z^	BBP^Y^ (%)	NSB^X^ (%)	DFE^W^ (d)	DEA^V^ (d)
Tre. 1	November 26, 2012	0 d^**U**^	2.14 c	65.33 a	—^**T**^
Tre. 2	December 10, 2012	0.19 c	2.48 bc	34.33 b	48.00 a
Tre. 3	December 24, 2012	0.62 b	3.19 b	27.00 c	45.33 b
Tre. 4	January 7, 2013	0.91 a	4.00 a	18.00 d	38.67 c
Tre. 5	January 21, 2013	1 a	4.19 a	13.33 de	17.33 d
Tre. 6	February 4, 2013	1 a	4.14 a	8.33 e	14.33 e
Tre. 7	February 25, 2013	1 a	4.47 a	—^**S**^	13.00 e
Tre. 8	March.11, 2013	1 a	4.24 a	—^**R**^	—^**R**^

^**Z**^The specific transfer time was 10 o’clock am in each transfer-date;

^**Y**^ **B**BP: **B**ud **B**reak **P**ercentage checked five weeks after being transferred into glasshouse;

^**X**^ **NSB**: average **N**umber of **S**prouted **B**uds per plant;

^**W**^ **DFE**: **D**ays number between the transfer-date and date of the **F**irst stem **E**longated visibly;

^**V**^ **DEA**: **D**ays number between the transfer-date and date of stem **E**longated visibly of **A**ll plants;

^**U**^Mean separation within columns by Duncan’s multiple range test at P = 0.01;

^**T**^Many plants of Tre. 1 never sprouted;

^**S**^The first visible stem elongation of Tre. 7 was occurred before being transferred into glasshouse;

^**R**^All plants of Tre. 8 sprouted and their stems elongated visibly before being transferred into glasshouse.

From the morphological observation, we can conclude the plants of Tre. 4 obtained enough chilling accumulation to ensure their all buds dormancy release, and the plants of Tre. 5 acquired more sufficient chilling to realize more rapid bud sprouting after temperature elevation. We assumed that Tre. 4 was the intermediate status beween Tre. 3 and 5, therefore, we did not use the buds from Tre. 4 for transcriptome sequencing. In addition, the differences of morphological indiecs were not significant between Tre. 7 and Tre. 8, so we also did not choose the sample from Tre. 8. Finally, we used the samples from Tre. 1 to 3, and Tre. 5 to 7, for subsequent transcriptome sequencing and analysis.

### Transcriptome sequencing and *de novo* assembly

The underground renewal buds were sampled from the plants of six selected treatments based on the morphological data, and assigned to six independent gene pools. After cleaning and performing quality checks, a total of 286,910,694 clean reads with an average size of 100 bp were obtained in all six pools (Table 2). Using the assembly method “Trinity”, all the sequences were assembled to 207,827 non-redundant unigenes with a mean length of 828.031 bp. The length of N50 was 1,473 bp, whereas the length of N90 was 313 bp. The percentage of Guanine and Cytosine (GC percentage in Table 2) was found to be 39.9%. Q20 percentage, which is an important indicator of assembly quality, and is defined as the percentage of sequences with sequencing error rate lower than 1%[47], was 99%. A total of 84,205 ORFs with average lengths of 785.561 bp were obtained, whereas 51,340 of them were complete and 10,254 were internal. The total numbers of ORFs with 5-prime partial and 3-prime partial were 15,344 and 7,267, respectively.

**Table 2:** Statistical summary of ‘Hangbaishao’ dormant bud transcriptome by Illumina RNA-Seq platform.

Items	Trinity	Trinity+PRICE
Total number of raw reads	294,792,058	294,792,058
Total number of clean reads	286,910,694	286,910,694
Average read length (bp)	100	100
Total nucleotides	294,792,058,000	294,792,058,000
Total number of unigenes	207,827	51,481
Average length of unigenes (bp)	828.031	1250.230
Max unigene bases	17,823	14,624
Min unigene bases	201	200
Total base pairs (bp)	172,087,126	64,363,071
Sequences with E-value < 10^-5^	61,603	31,950
Q20(%)	99	99
GC percentage(%)	39.9	39.7
N50	1,473	1,804
N90	313	597
Total number of ORF	84,205	32,739
Total number of ORF / Unigenes(%)	40.52	63.59
Average length of ORF (bp)	785.561	274.249
The number of complete ORF	51,340	21,315
The number of internal ORF	10,254	1,761
The number of ORF only with 5 prime partial	15,344	6,347
The number of ORF only with 3 prime partial	7,267	3,316

**Fig 3:**
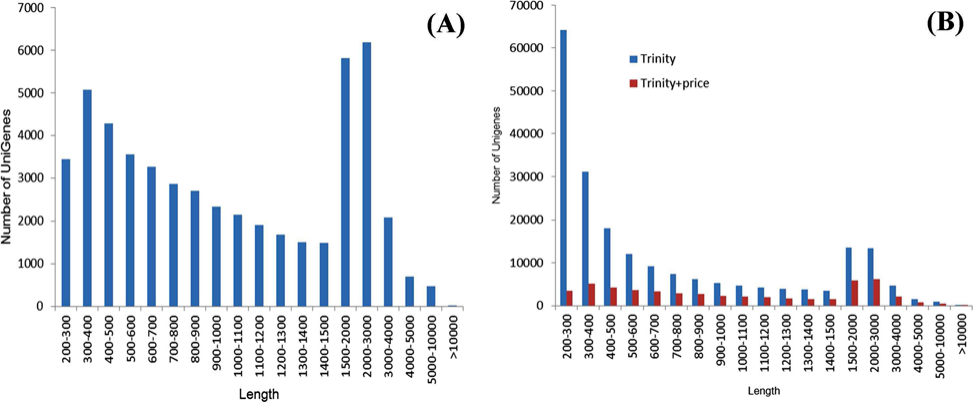
The length distribution (bp) of ‘Hangbaishao’ unigenes. **(A)** The length distribution of ‘Hangbaishao’ unigenes assembled by “Trinity+PRICE”. **(B)** Comparison of the length distribution of unigenes assembled by “Trinity” and “Trinity+PRICE”.

Trinity and PRICE were used in combination to assemble contigs generated by Trinity, and the unigenes obtained in this manner were more integrated than those assembled by Trinity alone (Table 2, Fig. 3). Although only 51,481 unigenes were generated (less than 207,827 of only “Trinity”), the mean length of 1,250.230 bp of these unigenes was much longer than 828.031 bp of the unigenes generated by “Trinity”. The lengths of N50 and N90 were 1,804 and 597, respectively, which were higher than those of “Trinity” (1,437 and 313, respecitvely). The lengths of 11,996 unigenes ranged from 1,500 bp to 3,000 bp, which accounted for 23.3% of the total. Only 13% of the unigenes obtained using “Trinity” had lengths in this range (Fig. 3B). In addition, a total of 38,703 and 9,473 unigenes were longer than 500 and 2,000 bp, which accounted for 75.2% and 18.4% of the total of 51,481 unigenes, respectively. In the transcriptome of dormant bud in garlic (*Allium sativum*), which is another important geophyte, only 16.23% (20,765/127,933) and 2.2% (2,793/127,933) unigenes assembled by SOAPdenovo program were longer than 500 and 2,000 bp, respectively [20,21].

The length of unigenes is crucial in determining the integrity of the assembly. The unigenes assembled by “Trinity+PRICE” had longer N50, N90, and mean lengths, so they are much longer and more integrated than the unigenes generated only by “Trinity” in our study, and the unigenes of garlic asssembled by SOAPdenovo program[20,21]. For these reasons, “Trinity+PRICE” is considered to be a better method for de novo assembly. The following transcriptome analysis in this paper were all based on the 51,481 unigenes assembled by the method “Trinity+PRICE”.

### Annotation of predicted proteins

**Fig 4:**
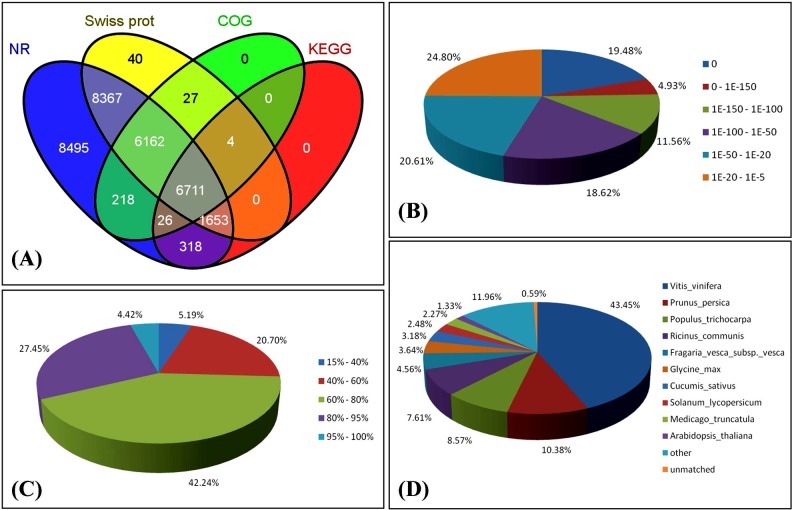
Characteristics of homology search of ‘Hangbaishao’ unigenes against the NR database. **(A)** Venn diagram of number of unigenes annotated by BLASTx with an E-value threshold of 10^-5^ against protein databases. The numbers in the circles indicate the number of unigenes annotated by single or multiple databases. **(B)** E-value distribution of the top BLASTx hits for each unigene. **(C)** Similarity distribution of the top BLASTx hits for each unigene. **(D)** Species distribution of the top BLASTx hits for all homologous sequences.

The percentage of 62.77% unigenes (32,316/51,481) matched significantly with known genes in public databases (Table 3), which are much higher than 36.81% (47,095/127,933) in unigenes annotation of garlic[20,21]. The key reason is probably that our unigenes are more integrated than the garlic unigenes, and partial unigenes of garlic were too short to allow statistically meaningful matches[20]. Among these data in the present study, 31,950 unigenes were annotated against NR, whereas only 8,712 unigenes were annotated to the KEGG database (Table 3, Fig. 4A). “Non-BLASTable” sequences have been reported for all studied plant transcriptomes, and their proportion depends on the species, sequencing depth, and parameters of BLAST search. Besides the technical issues derived from sequencing, biological factors may be responsible for the large population of non-BLASTable sequences, including species-specific genes, lack of genome and the persistence of non-coding fractions mainly from untranslated regions of the sampled transcripts[48,49].

**Table 3:** BLAST analysis results of ‘Hangbaishao’ against five public databases.

Database	Number of annotated unigenes	Percentage of annotated unigenes (%)
NR	31,950	62.06
GO	22,775	44.24
KEGG	8,712	16.92
Swiss-prot	22,964	44.61
COG	13,148	25.54
Total	32,316	62.77

The characteristics of the homology of Illumina sequences against the NR were further analyzed. Based on the NR annotations, 54.59% of the annotated sequences had very strong homologies (E-value < 10^-50^), 20.61% showed strong homologies (10^-50^ < E-value < 10^-20^), and an additional 24.80% showed weak homologies (10^-20^ < E-value < 10^-5^) (Fig. 4B). The similarity distribution was comparable, with 31.87% of the sequences having similarities higher than 80%, whereas 42.24% of the hits had similarities of 60% to 80% (Fig. 4C). The top-hit species was *Vitis vinifera* with a hit of 43.45% for 13,882 assembled sequences (Fig. 4D). Approximately 10.83% of the sequences were mapped to *Prunus persica*, whereas 1% to 9% of orthologous genes were mapped to *Populus trichocarpa*, *Ricinus communis*, and *Fragaria vesca* subsp. *vesca*, etc. Moreover, 187 unigenes (0.59%) were not mapped to any species.

The region of coding sequences (CDS) are identified as a sequence of nucleotides that corresponds with the sequence of amino acids in a protein. A coding sequence region is bounded at the 5’ end by a start codon and at the 3’ end with a stop codon. CDS can be a subset of an ORF, therefore, CDS identification is an important part of unigenes prediction[50], and has been performed in many studies on plants and animals[43,44,47,49,50]. BLASTx was used against the databases mentioned in this study, and 32,739 CDS (63.59% of 51,481 unigenes) were translated into peptide sequences, in which 10,033 (30.65% of CDS) had lengths greater than 300 AA, with the 683 longest unigenes having lengths over 1,000 AA. All CDS were translated into amino sequences with the standard codon table. Details on length and amino sequences are shown in S1 File. These data can provide a useful reference for future studies on gene structure and function.

### Functional classification

#### COG classification

**Fig 5:**
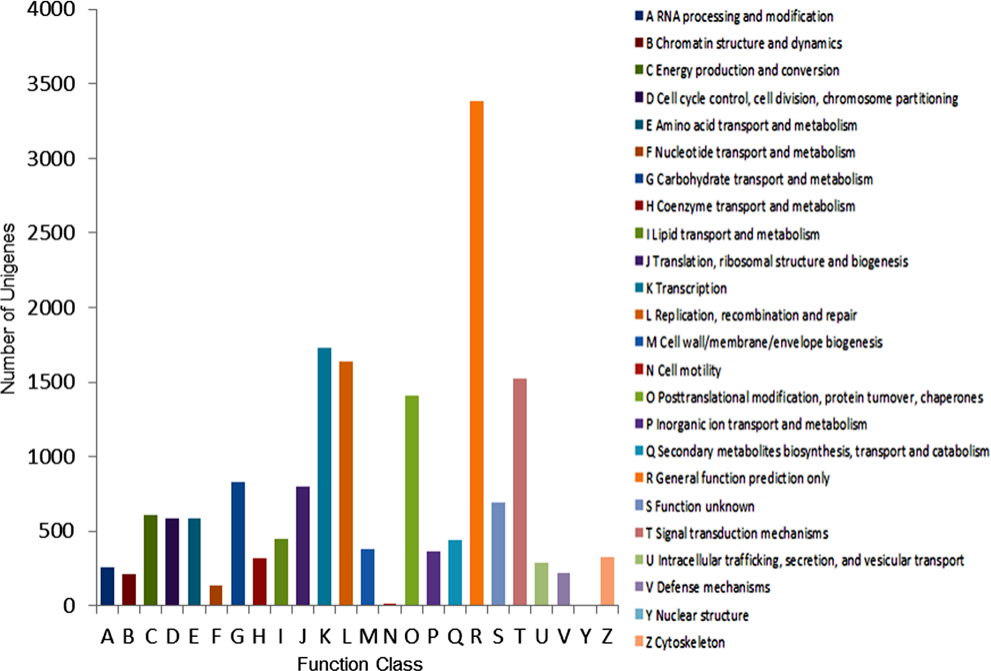
COG function classification of ‘Hangbaishao’ unigenes.

In our study on *P*. *lactiflora*, unigenes were aligned to the COG database to predict and classify possible functions. A total of 17,176 out of 51,481 sequences had COG functional classifications, which were grouped into 24 functional categories (Fig. 5). The single most common category was “General function prediction” (group R) with 3,382 unigenes, followed by “Transcription” (1,727, group K), “Replication, recombination, and repair” (1,637, group L), “Signal transduction mechanisms” (1,527, group T) and “Posttranslational modification, protein turnover, and chaperones” (1,407, group O). “Nuclear structure” (6, group Y) and “Cell motility” (14, group N) were the smallest COG categories. In addition, a total of 827, 606, and 583 unigenes were annotated to the categories of “Carbohydrate transport and metabolism” (group G), “Energy production and conversion” (group C) and “Cell cycle control, cell division, and chromosome partitioning” (group D), respectively. All of these categories are related to dormancy release and bud breaking.

#### GO classification

**Fig 6:**
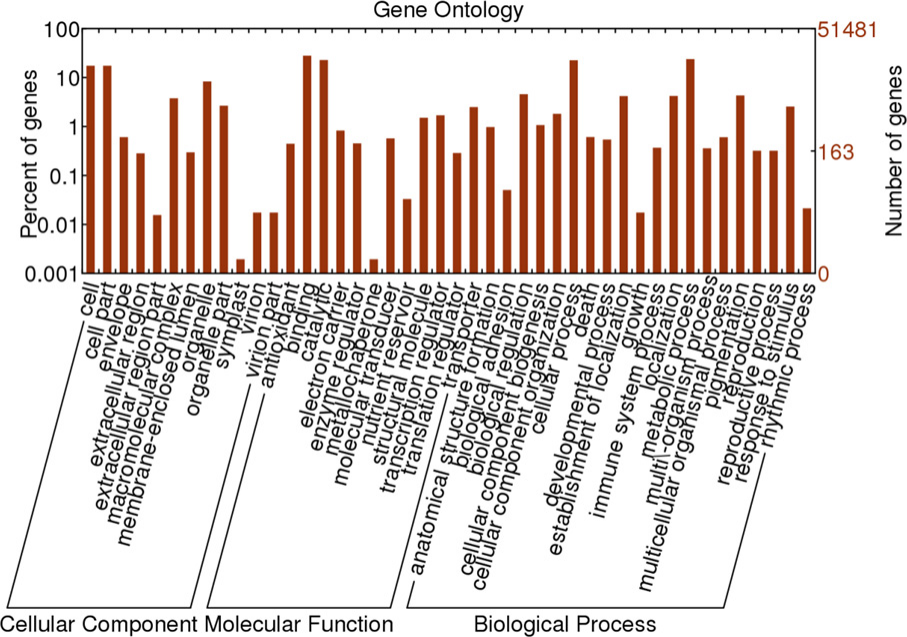
GO classification of ‘Hangbaishao’ unigenes.

The GO classification can provide a controlled vocabulary to facilitate high-quality functional gene annotations[51]. A total of 45,598 out of 51,481 unigenes (88.57%) with GO annotation were classified based on “Biological process”, “Cellular component”, and “Molecular function” (Fig. 6). In all, 9,089 sequences were assigned to the “Cellular component” ontology, 20,511 were assigned to “Molecular function” and 15,998 sequences were assigned to “Biological process” ontology. The most well-represented cellular components were “cell” and “cell parts”, both of which accounted for 23.9%. By contrast, the well-represented cellular components of garlic were “plastid” and “mitochondrion”[20,21]. In terms of molecular functions of ‘Hangbaishao’, “catalytic activity” and “binding” were the two best-represented functions with percentages of 49.3% and 54.3%. In garlic, the well-represented molecular functions were “transferase activity” and “nucleotide binding”, respectively[20,21]. In the biological process category of ‘Hangbaishao’, 50.0% and 40.8% were related to “metabolic process” and “cellular processes”, respectively. However, “protein metabolic process” and “localization” were the most well-represented in garlic[20,21]. We cannot explain these differences of GO classification between herb peony and garlic. In addition, we noticed that “hydrolase activity” and “carbohydrate metabolic process” represented well in garlic, but did not appear on GO classification of ‘Hangbaishao’. The buds and bulbs of garlic are rich in starch, the mutual transformations of starch and soluble sugar occur during the garlic bud dormancy induction, transition or release. Therefore, it is not strange that many genes were classified into “hydrolase activity” (such as starch hydrolytic enzyme activity) and “carbohydrate metabolic process” of GO classification in garlic transcriptome.

A large number of unigenes was assorted to the “cell”, “cell parts”, “cellular processes” and “metabolic processes” categories, thus we speculated that abundant unigenes were closely related to cell life, division and growth, and changed significantly during dormancy release and bud breaking of ‘Hangbaishao’. This speculation has been confirmed in other dormancy studies of shrubs or perennials, such as *Vitis riparia*[14], *P*. *ostii*[30] and *Euphorbia esula*[52,53]. A total of 229 unigenes were involved in antioxidant activity. Oxidative stress is believed to occur in bud dormancy and hinders the buds from breaking when plants are exposed to severe cold. Antioxidant enzymes can eliminate the threats of increased oxidative stress in buds under low temperature[30], and may help to promote sprouting[54,55]. The changes in biochemical levels of two antioxidant enzyme activities (POD and SOD) will be analyzed later. In addition, 2,219 unigenes were assigned to the subcategory of pigmentation and are possibly related to the deposition of coloring matter in pink purple, pink, and pink white petals of *P*. *lactiflora* ‘Hangbaishao’[48].

### Transcription factors and transcripts

#### Transcription factor families

Transcription factors (TFs) have important functions in gene expression regulation during plant development and responses to environmental stress. Identification, comparison, and analysis of the TFs can be helpful in understanding their functions in the bud dormancy of ‘Hangbaishao’. Based on our BLAST data, abundant proteins could be identified as putative TFs or regulators belonging to different families, such as basic helix-loop-helix protein (bHLH), myeloblastosis viral oncogene homolog B (MYB), ethylene response factor (ERF), auxin response factor (ARF), WRKY and APETALA2 (AP2) (Table 4).

**Table 4:** Transcription factor families identified from ‘Hangbaishao’ transcriptome libraries.

Putative transcription factor family	Number of unique transcripts	Putative transcription factor family	Number of unique transcripts
bHLH	115	HD-ZIP	42
MYB	114	Nin-like	35
FAR1	61	MYB-related	35
SBP	59	B3	35
ERF	59	AP2	33
C2H2	57	G2-like	27
ARF	55	GRAS	26
bZIP	52	MIKC	0
NAC	51	Others	299
WRKY	50	Total No. of TFs	1251
C3H	46		

The largest number of unique transcripts was observed in the bHLH TF family (115 unique transcripts), which has a key function in hormone homeostasis, light signaling[56], proanthocyanidin biosynthesis[57] and responses to various biotic and abiotic stresses. Taking the cold tolerance response as an example, the apple bHLH gene *MdCIbHLH1* (cold-induced bHLH1) is remarkably induced in response to cold stress, and its overexpression enhances cold tolerance in transgenic *Arabidopsis*[58]. Gene expression profiles revealed that *MabHLH1/2/4*, isolated and characterized from banana fruit, is also induced by cold stress[59]. Based on these, we speculate that transcripts of the bHLH family are induced by low temperature and accumulate to increase the chilling tolerance of ‘Hangbaishao’ during its dormancy. ndf ms and leaves from The second largest family was the MYB TFs family (114 unique transcripts), which is an important cold-inducible transcription factor family[60]. The MYB TFs family has also been identified in the dormant buds of tree peony (more abundant than the other TFs)[29]. The MYB TFs family has obvious functions in anthocyanin biosynthesis[61] and resistance to abiotic stress, such as dryness[62], cold[60,63] and salt stress[64]. However, the precise function of the MYB family in abiotic stress response is still not fully understood.

The sequences belonging to the TFs families of bZIP, ERF, WRKY and NAC were also relatively abundant in the present study. These families are easily triggered by oxidative signals and configure the early response mechanisms to chilling stress[65]. The MADS-box family, which has a dominant function in plant floral development[29,66], was not found in the present study, probably because floral formation was done prior to temperature fall in autumn. Only nine transcripts of MADS-box IFs were identified in the previous study on *P*. *ostii*[29]. In the Japanese apricot, MADS-box genes were repressed by prolonged chilling exposure[67]. The MADS-box TF (DAM) family in *P*. *mume* was found to be closely associated with dormancy induction and release[18].

#### Highly expressed transcripts

By removing the unigenes that were not annotated in NR of some representative plant species, the ten most highly expressed unigenes of the *P*. *lactiflora* transcriptome libraries were identified (Table 5). Five of these ten unigenes (Unigene 028024, Unigene 048324, Unigene 022315, Unigene 033567 and Unigene 022968) were annotated as DHN by NR from sequences of *Malus* × *domestica*, *Corylus heterophylla* and *Phaseolus vulgaris*. DHNs comprise one of the plant protein families typically induced by multiple stresses that cause cellular dehydration, and protect plant proteins and membranes from damage[68,69]. *BjDHN2* and *BjDHN3* exert a protective function on membranes under abiotic stresses, both of which are upregulated by low temperature, drought and salt stress in *Brassica juncea*[68]. Similar findings were obtained in *Gentiana triflora*: transgenic gentian plantlets overexpressing *GtDHN1* or *GtDHN2* showed improved cold and drought stress tolerance. Accumulation of GtDHNs may be part of the strategy for winter survival of overwintering buds in *Gentiana triflora*[70]. In *Medicago sativa*, DHNs have a key function with regard to tolerance to subfreezing temperatures[71].

**Table 5:** The ten most highly expressed transcripts in ‘Hangbaishao’ transcriptome libraries.

Gene ID	NR ID	BLAST annotation
Unigene028024	gi|382948197|gb|AFG33213.1|	dehydrin 3 [*Malus* × *domestica*]
Unigene048324	gi|307776652|gb|ADN93460.1|	dehydrin 2 [*Corylus heterophylla*]
Unigene039066	gi|15149875|emb|CAC39481.2|	metallothionein-like protein [*Quercus suber*]
Unigene022315	gi|307776652|gb|ADN93460.1|	dehydrin 2 [*Corylus heterophylla*]
Unigene030667	gi|462394450|gb|EMJ00249.1|	hypothetical protein PRUPE ppa018344mg [*Prunus persica*]
Unigene034306	gi|147779298|emb|CAN76800.1|	hypothetical protein VITISV 043026 [*Vitis vinifera*]
Unigene051377	gi|284437887|gb|ADB85571.1|	D-galacturonic acid reductase 1 [*Actinidia deliciosa*]
Unigene040905	gi|158564580|gb|ABW74478.1|	unknown [*Paeonia suffruticosa*]
Unigene033567	gi|1326161|gb|AAB00554.1|	dehydrin [*Phaseolus vulgaris*]
Unigene022968	gi|307776652|gb|ADN93460.1|	dehydrin 2 [*Corylus heterophylla*]

Unigene 039066 was annotated as a metallothionein-like protein based on the sequence of *Quercus suber*, and may be regulated by multiple abiotic stresses[72,73]. Unigene 051377 was identified as D-galacturonic acid reductase from the sequence of *Actinidia deliciosa*. Overexpression of D-galacturonic acid reductase enhances tolerance to various abiotic stresses in transgenic potato[74]. Based on these analyses, we found that DHNs, metallothionein-like protein, and D-galacturonic acid reductase were all related to abiotic stress. Unigene 040905 was annotated to the sequence of *P*. *suffruticosa*, which belongs to the same genus as *P*. *lactiflora*. However, the function of this unigene remains unclear.

### SSR identification

SSRs are highly popular genetic markers because of their high abundance, hypervariability, extent of allelic diversity, co-dominant inheritance, and suitability for high-throughput analysis[44,75,76]. Approximately 17,705 SSRs (34.39% of 51,481 examined unigenes) were generated and distributed in 13,797 sequences (Table 6). A total of 3,103 sequences contained more than one SSR; 1,182 of identified SSRs were found in compound formation, whereas others were of the perfect one-repeat type. The percentages of SSR motifs, as well as types and frequencies of major repeats, were shown in Table 7. Besides mononucleotides (11,119; 62.80%), the most two common repeat motifs were dinucleotides (4,518; 25.52%) and trinucleotides (1,892; 10.69%). In comparison, there were extremely few repeat motifs that were tetranucleotides (105; 0.59%), pentanucleotides (25; 0.14%) and hexanucleotides (46; 0.26%). An obvious motif bias was found for AG, AAG, and AAAT of di-, tri-, and tetranucleotides.

**Table 6:** General statistics of SSR search in ‘Hangbaishao’ transcriptome libraries.

Source	Number
Total number of sequences examined	51,481
Total size of examined sequences (bp)	64,414,551
Total number of identified SSRs	17,705
Number of SSR containing sequences	13,797
Number of sequences containing more than one SSR	3,103
Number of SSRs present in compound formation	1,182

**Table 7:** General statistics of repeat type of SSR motif from ‘Hangbaishao’ transcriptome libraries.

SSR motif	Number	Percentage (%)	Major repeat type and frequency
Mononucleotides	11119	62.80	A/T(61.80%)
Dinucleotides	4518	25.52	AG/CT(16.51%)
Trinucleotides	1892	10.69	AAG/CTT(2.26%)
Tetranucleotides	105	0.59	AAAT/ATTT(0.27%)
Pentanucleotides	25	0.14	AGAGG/CCTCT(0.07%)
Hexanucleotides	46	0.26	AGCCTC/AGGCTG(0.06%)

### Physiological and biochemical pathways

#### KEGG categories and annotations

KEGG analysis provides an alternative functional annotation based on unigenes associated with biochemical pathways. Overall, 5,129 unigenes were mapped to the KEGG database, accounting for 9.96% of the total. A total of 251 inner-cell metabolic pathways were predicted (S1 Table). Maps with the highest unigene representation were metabolic pathways (KO 01100; 1,660 unigenes, 32.36%), followed by pathways for the biosynthesis of secondary metabolites (KO 01110; 859 unigenes, 16.75%), microbial metabolism in diverse environments (KO 01120; 400 unigenes, 7.80%), spliceosomes (KO 03040; 321 unigenes, 6.26%), and protein processing in the endoplasmic reticulum (KO 04141; 274 unigenes, 5.34%). We found that 1,934 unigenes associated with carbohydrate metabolism in KEGG biochemical pathway annotation of garlic transcriptom[20,21]. However, carbohydrate metabolism did not appear in the present KEGG pathway of ‘Hangbaishao’, and only 7 unigenes (0.14% of the total) were annotated as carbohydrate digestion and absorption (S1 Table). The reason of this difference is probably the same as the cause of that many genes were classified into hydrolase activity and carbohydrate metabolic process of GO classification in garlic transcriptome research mentioned before.

**Fig 7:**
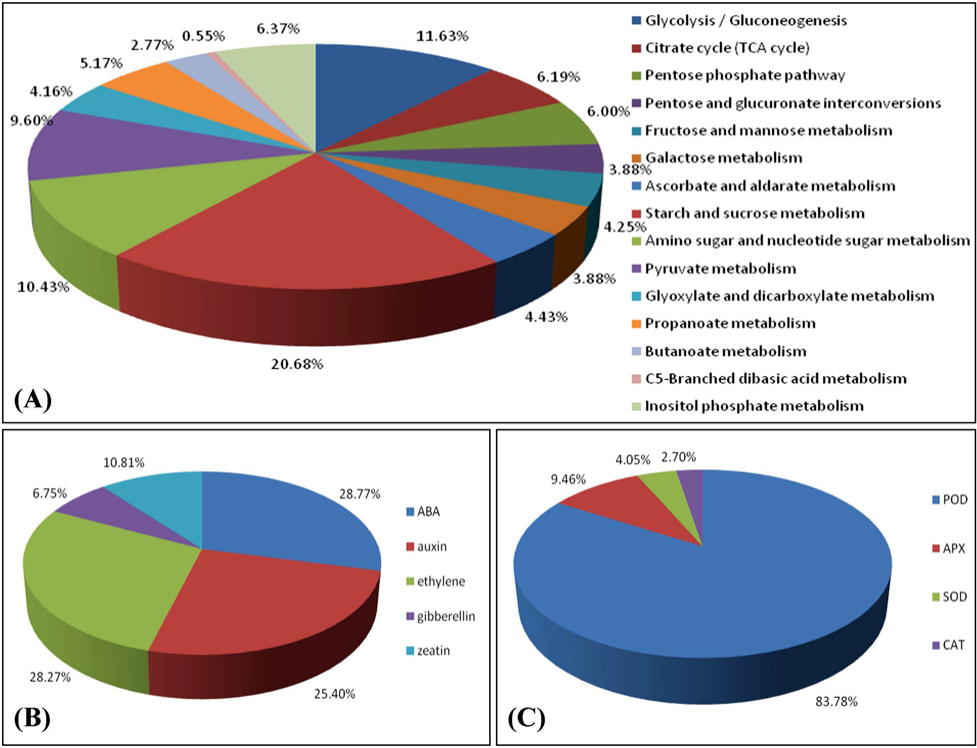
The proportions of sequences related to carbohydrate metabolism, plant hormones and antioxidant enzymes. **(A)** The proportion of sequences related to carbohydrate metabolism. **(B)** The proportion of sequences related to five internal plant hormones. **(C)** The proportion of sequences related to four antioxidant enzymes.

We focused on some key pathways involved in the bud dormancy transition and release, including carbohydrate metabolism, plant hormones and antioxidant response-related pathways. A total of 1,083 unigenes were associated with carbohydrate metabolism according to KEGG. These unigenes could be subdivided further into genes involved in starch and sucrose metabolism, glycolysis/gluconeogenesis, pyruvate metabolism, and so on. (Fig. 7A). The processes involved in the starch and sucrose metabolic pathways, the important carbohydrate metabolic pathways, accounted for 20.68% (224/1082) of the total. Based on the results of BLAST analysis, 47 key enzymes out of 91 enzymes defined by 224 unigenes possibly encode enzymes involved in starch and sucrose metabolism (EC numbers are noted in green boxes in S1 Fig.).

Plant hormones play important roles in dormancy release and budbreak. In this study, 1,008 unigenes were related to five hormones according to KEGG (Fig. 7B). Further analysis showed that 28.77%, 25.40%, and 28.27% were associated with ABA, auxin, and ethylene, respectively, which together comprised the largest proportion of the five hormones. Only 68 and 109 unigenes were related to gibberellic acid (GA) and zeatin, respectively.

Recent studies have indicated that oxidative stress accompanies the response to dormancy breaking in horticultural crops. Antioxidant enzymes can be produced when crops suffer from cold stress[60]. They can eliminate increased oxidative stress in buds during chilling treatment and promote bud breaking[11,30,54,55]. In this study, 74 unigenes related to four important antioxidant enzymes (Fig. 7C), which are POD, ascorbate peroxidase (APX), CAT, and superoxide dismutase (SOD), respectively, were mapped into the KEGG database. Approximately 62 unigenes were related to POD, accounting for 83.78% of the total, which was higher than three other antioxidant enzymes.

#### Dormancy biochemical observation

We specially focused on the content changes of IAA and ABA, and dynamic activities of POD and CAT. As shown in Table 8, similar dynamic trends were observed between the contents of ABA and IAA, although their trends have been reported to be usually opposite in many previous studies[26,39]. Endogenous ABA has been confirmed to facilitate cold acclimation and maintain dormancy[77], and is assumed to be not associated with dormancy release[8,78,79]. On the other hand, IAA has been known to have significant effect on dormancy release and bud sprouting[80]. We cannot explain the reason behind the similar trends observed between IAA and ABA in this study, but we believe cross-talk between ABA and IAA can better reflect the progress of dormancy release. We calculated the ratio of IAA to ABA content (IAA/ABA) and found that it increased sharply after February 4, 2013 (the sampling time of Tre. 6), confirmed that the increase in IAA content and the decrease in ABA content were closely related to the division and growth of meristematic cells in the underground buds, and promoted rapid extension of the buds.

**Table 8:** Biochemical observation for the underground renewal buds during ‘Hangbaishao’ bud dormancy.

Treatment	IAA[ng (g FW)^-1^]	ABA[ng (g FW)^-1^]	IAA/ABA	POD(μmol mg^-1^ protein min^-1^)	CAT(μmol mg^-1^ protein min^-1^)
Tre. 1	33.85f^Z^ ±0.15	83.88e ±1.18	0.40	0.96c±0.10	0.63d±0.08
Tre. 2	62.41b ±0.54	113.89b ±0.94	0.55	1.00c±0.02	0.92c±0.03
Tre. 3	46.56d ±0.26	101.07d ±0.65	0.46	0.97c±0.09	1.51a±0.01
Tre. 4	49.23c ±0.14	107.39c ±0.56	0.46	1.34b±0.07	0.95c±0.08
Tre. 5	63.65b ±0.62	110.33bc ±1.07	0.58	1.31b±0.04	1.25b±0.05
Tre. 6	43.63e ±0.19	118.60a ±1.47	0.37	1.64a±0.10	1.07bc±0.03
Tre. 7	44.91de±0.32	72.94f ±0.20	0.62	1.74a±0.04	1.25b±0.10
Tre. 8	120.75a ±1.06	121.77a ±1.04	0.99	0.73d±0.08	1.19b±0.09

^Z^Mean separation within columns by Duncan’s multiple range test at P = 0.01

Research so far on antioxidant enzyme activities during the bud dormancy have mainly been carried out in fruit trees, such as grape[55], peach[81] and lemon[82], or some macrophanerophytes with important uses for the economy or afforestation, such as beech[83] and pine tree[84]. Comparatively, the studies on antioxidant enzyme activities have been relatively limited in the dormant buds of geophytes[80,85–88]. In the present study, we observed that CAT activity fluctuated, but exhibited a gradual increase overall (Table 8), which plays an important role in protection of organs and tissues under stress and promotion of bud dormancy release[54,83,84]. POD is another important antioxidant enzyme, and its activity rose steadily during the first seven phases, and decreased rapidly in the last phase. Some studies on geophytes have reported that POD activity usually increased during bud dormancy release before sprouting, and decreased when buds were elongating and sprouting[80,87]. The dynamic change in POD activity observed in our study were consistent with this change law overall. POD is closely involved in hydrogen peroxide metabolism in response to various environmental stresses. Increased POD activity could eliminate the javascript:void(0);free radicals accumulated under low temperature, and alleviate increased oxidative stress, thereby protecting the dormant buds from cold injury[85] and promoting bud elongation and breaking[54]. Similar results have been reported in studies on fruit trees; for instance, expression levels of peroxidase RNA transcripts were found to be obviously enhanced in grapevine buds under low temperature[89].

Some interesting correlations are noteworthy between hormone contents and antioxidant enzyme activities. For instance, compared with the drastic rise in IAA between the phase of Tre. 7 and 8, POD activity reduced sharply and in that phase the buds elongated obviously (Table 8). The POD function has been widely researched, especially in relation to its catalytic activity on IAA, which is generally considered as an effective promoter of bud dormancy release and sprouting[80]. We suspected that during the period from Tre.1 to 7, the prolonged increase in POD activity may lay the foundation for surge in IAA content in the last phase. When the IAA content rose drastically in the eighth phase, correspondingly, POD activity decreased sharply. This is only a hypothesis, and both of the correlations between change in POD activity and IAA content, and the specific reason for this correlation should be subjects of future research.

### Dormancy status identification

**Fig 8:**
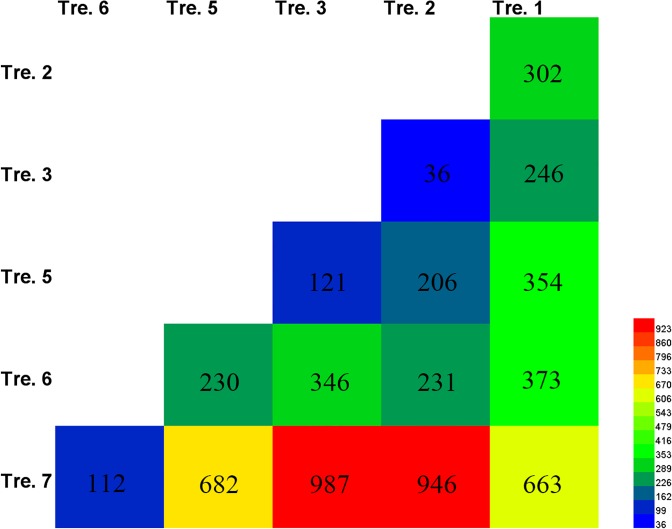
The number of DEGs derived from 15 pairwise comparisons of six treatments.

The analysis on the differences in the number of DEGs between any two of six independent gene pools (Fig. 8) in combination with morphological and biochemical observations (Tables 1 and 8), could contribute to estimate the dormancy status of the underground renewal buds in *P*. *lactiflora* ‘Hangbaishao’, A total of 2,057 DEGs were identified after 15 pairwise comparisons. The number of DEGs was 302 in the Tre. 1-VS-Tre. 2 comparison, which is much more than those in the Tre. 2-VS-Tre. 3 (36), Tre. 3-VS-Tre. 5 (121), Tre. 5-VS-Tre. 6 (230) and Tre. 6-VS-Tre. 7 (112) comparisons (Fig. 8). This difference means the bud dormancy status in the first phase was remarkably different with those in other four phases. As shown in Table 1, four morphological indices revealed that chilling accumulation of the buds in Tre. 1 was too unsufficient to break dormancy, whereas since Tre. 2 all plants of the latter seven treatments could sprout although their DEA were different. Based on these results, we assumed the underground buds were probably still in the endodormant stage on November 26, 2012 (the sampling time of Tre. 1); and thereafter, the transition from endodormancy to ecodormancy occurred between November 26 and December 10, 2012 (the time of Tre. 2).

The dormancy status of the buds in Tre. 7 was also distinctly different with other treatments except for Tre. 6, because relatively more DEGs were identified in the Tre. 1-VS-Tre. 7 (663), Tre. 2-VS-Tre. 7 (946), Tre. 3-VS-Tre. 7 (987) and Tre. 5-VS-Tre. 7 (682) comparisons (Fig. 8). Actually, most buds elongated and thickened visibly on February 25, 2013 (the time of Tre. 7) before the plants being transferred into greenhouse; meanwhile, the ratio of IAA to ABA content rose significantly in Tre. 7 (Table 8). Therefore, we believed that ‘Hangbaishao’ buds have finished the release from ecodormancy and entered the stage of bud elongation and thickening on February 25, 2013.

The bud dormancy status of Tre. 6 might be similar with that in Tre. 7 instead of Tre. 5, because the number of DEGs in the Tre. 6-VS-Tre. 7 comparison (112) was much less than that in the Tre. 5-VS-Tre. 6 comparison (230) (Fig. 8). But the buds did not yet elongate obviously on February 4, 2013 (the time of Tre. 6). The possible reason is that the buds of Tre. 6 were still in the stage of ecodormancy release (the end of ecodormancy and initial stage of bud elongation, thickening and sprouting).

In conclusion, combined with the data on the number of DEGs and dormancy morphology and physiology, we assumed that ‘Hangbaishao’ buds were in the endodormant stage before November 26, 2012 (Tre. 1); and next in the transition stage from endo- to eco-dormancy between November 26 (Tre. 1) and December 10, 2012 (Tre. 2); then in the ecodormant stage during the period from December 10, 2012 (Tre. 2) to January 21, 2013 (Tre. 5); and in the stage of release from ecodormancy between January 21 (Tre. 5) and February 25, 2013 (Tre. 7); and finally in the stage of bud elongation, thickening and sprouting after February 25 (Tre. 7) of course also including March 11, 2013 (Tre. 8) (Table 9).

**Table 9:** Bud dormancy status of ‘Hangbaishao’ in different periods.

Period	Treatment	Dormancy status
**≤** ^**Z**^ November 26, 2012	**≤** Tre. 1	Endodormancy
>^**Y**^ November 26, 2012 and <^**X**^ December 10, 2012	> Tre. 1 and < Tre. 2	Transition from endodormancy to ecodormancy
**≥** ^**W**^ December 10, 2012 and **≤** January 21, 2013	**≥** Tre. 2 and **≤** Tre.5	Ecodormancy
> January 21 and < February 25, 2013	> Tre. 5 and < 7	Ecodormancy release
**≥** February 25, 2013	**≥** Tre. 7	Bud elongation, thickening and sprouting

^Z^The symbol “≤” means this dormancy status occurred before this treatment-day and also in this treatment-day;

^Y^The symbol “>” means this dormancy status occurred after this treatment-day but did not in this treatment-day;

^X^The symbol “<” means this dormancy status occurred before this treatment-day but did not in this treatment-day;

^W^The symbol “≥” means this dormancy status occurred after this treatment-day and also in this treatment-day.

### Main physiological or biochemical metabolisms in five dormant phases identified by KEGG pathway annotation

In order to discover more new biochemical insights during the bud dormancy transition and release, further study on the inner metabolic changes of buds should be conducted based on transcriptome level. As shown by Table 10 and S2 Table, we could obtain the profile of physiological or biochemical metablisms during the five adjacent dormant phases based on KEGG pathway annotation.

**Table 10:** DEGs and main physiologcal or biochemical metabolisms identified by KEGG pathway annotation during five adjacent dormant phases.

Pairwise comparisons	Number of DEGs (ND)^Z^	Number of DEGs annotated by KEGG pathway (NDK)^Y^	Percentage of NDK/ND (%)	Number of up-regulated DKs^X^	Number of down-regulated DKs
Tre. 1-VS-Tre. 2	302	36	12	18	18
Tre. 2-VS-Tre. 3	36	1	3	1	0
Tre. 3-VS-Tre. 5	121	18	15	18	0
Tre. 5-VS-Tre. 6	230	26	11	15	11
Tre. 6-VS-Tre. 7	112	20	18	20	0
	**Main physiologcal or biochemical metabolisms and up- or down-regulation of DEGs annotated as these pathways**				
Tre. 1-VS-Tre. 2	Glutathione metabolism(↑)^**W**^; Metabolism of xenobiotics by cytochrome P450(↑); Starch and sucrose metabolism(↑); Drug metabolism—cytochrome P450(↑); Protein processing in endoplasmic reticulum(↑); DNA replication(↓)^**V**^; Cell cycle(↓); Cell cycle—yeast(↓); Meiosis—yeast(↓); Metabolic pathways(↓); Biosynthesis of secondary metabolites(↓)				
Tre. 2-VS-Tre. 3	alpha-Linolenic acid metabolism(↑); Metabolic pathways(↑)				
Tre. 3-VS-Tre. 5	Phenylalanine metabolism(↑); Methane metabolism(↑); Phenylpropanoid biosynthesis(↑); Metabolic pathways(↑); Biosynthesis of secondary metabolites(↑); Steroid biosynthesis(↑); Biosynthesis of secondary metabolites(↑)				
Tre. 5-VS-Tre. 6	Protein processing in endoplasmic reticulum(↑); Cell cycle(↑); Cell cycle—yeast(↑); Meiosis—yeast(↑); ABC transporters(↓); Biosynthesis of secondary metabolites (↓)				
Tre. 6-VS-Tre. 7	Glutathione metabolism(↑); Metabolism of xenobiotics by cytochrome P450(↑); Drug metabolism—cytochrome P450(↑); Cell cycle(↑); Plant hormone signal transduction(↑); Progesterone-mediated oocyte maturatio(↑); Metabolic pathways(↑); alpha-Linolenic acid metabolism(↑)				

**^Z^ND**: **N**umber of **D**EGs;

**^Y^NDK**: **N**umber of **D**EGs annotated by **K**EGG pathway;

**^X^DKs**: **D**EGs annotated by **K**EGG pathway;

^W^The symble “↑” means the DEGs annotated as this pathway were mainly up-regulated;

^V^The symble “↓” means the DEGs annotated as this pathway were mainly down-regulated.

The first half of Table 10 showed the summary of DEGs annotated by KEGG pathway. The percentage of NDK/ND is not high, only 3 to 18%. The number of up-regulated DKs is more than the down-regulated ones in most phases. In the latter part of this table, we can find that main metabolic pathways were glutathione metabolism, metabolism of xenobiotics by cytochrome P450, starch and sucrose metabolism, etc. in the phase of Tre. 1-VS-Tre. 2 (means the phase between the sampling time of Tre. 1 to 2, i.e. November 26, 2012 to December 10, 2012). Dormancy transition occurred in this phase, the underground buds were still in relatively quiescent status under cold stress. Therefore, it is reasonable that three DEGs (unigene016230, unigene044767 and unigene030347) were down-regulated which annotated as DNA replication, cell cycle, cell cycle—yeast and meiosis—yeast in this phase (Table 10; sheet 1 of S2 Table). On the contrary, in the phase of Tre. 5-VS-Tre. 6, four DEGs (unigene004439, unigene016230, unigene030347 and unigene001283) which were also annotated as DNA replication, cell cycle, cell cycle—yeast or meiosis—yeast, were up-regulated before bud elongated visibly (Table 10; sheet 4 of S2 Table). Among these DEGs, unigene016230 and 030347 were twice annotated as these four pathways in both two phases, so these two genes may play key roles in the regulation of cell division or elongation and bud growth.

Only one DEG (unigene023828) was annotated by KEGG pathway in the phase of Tre. 2-VS-Tre. 3, which was related to alpha-linolenic acid metabolism and metabolic pathways (Table 10; sheet 2 of S2 Table). The annotated DEGs were all up-regulated in the phases of Tre. 3-VS-Tre. 5 and Tre. 6-VS-Tre. 7 (Table 10; sheets 3 and 5 of S2 Table). The pathways of glutathione metabolism, cell cycle and plant hormone signal transduction are closely associated with dormancy release, plant growth and tolerance to environmental stress[90], which became the main pathways in the last Tre. 6-VS-Tre. 7 phase (Table 10; sheet 5 of S2 Table).

### Expression of unigenes related to bud dormancy transition or release

**Fig 9:**
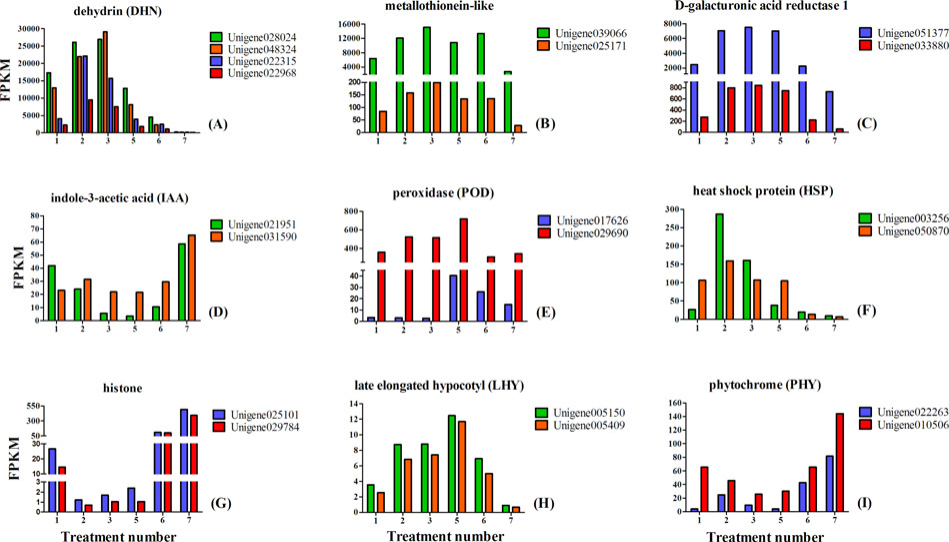
Expression of 20 interested unigenes related to the bud dormancy transition or release.

We chose 20 interested unigenes (most of which were DEGs) and showed their expression patterns by FPKM value in Fig. 9. Based on the annotations of NR and KEGG pathway, etc., these unigenes were determined to be some factors related to the bud dormancy transition or release, which were DHN, metallothionein, D-galacturonic acid reductase 1, IAA, POD, HSP, histone, LHY and PHY (S3 Table).

### Genes annotated as DHN, metallothione and D-galacturonic acid reductase 1

Eight unigenes were annotated as DHN, metallothionein and D-galacturonic acid reductase 1, and of which six genes highly expressed and entered the top 10 list of expression levels of all 51,481 unigenes (unigene028024, unigene048324, unigene022315, unigene022968, unigene039066 and unigene051377 in Table 5; Fig. 9A, Fig. 9B and Fig. 9C; S3 Table). We were interested in these genes with extremely high expression levels, and found most of which reached the peak of FPKM values in the sampling time of Tre. 3 (December 24, 2012), and after that their FPKM values decreased. As shown in Fig. 2, temperature fell steadily before Tre. 3 and rose gradually after Tre. 4. Therefore, the trend of temperature was basically opposite to the expression changes of these eight genes. As analyzed in the part of “Highly expressed transcripts”, six transcripts annotated as DHN, metallothione and D-galacturonic acid reductase 1 (Table 5) were closely related to various environmental stresses, especially the DHN genes were easily inducible under low temperature[91]. Based on these studies, we believed these eight genes may act as roles to provide protection for dormant buds under the cold, thereby led to their up-regulation before Tre. 3 and down-regulation when the temperature elevated[12]. Some researches reported that the proteins accumulated under the cold or drought stress were known as roles of stabilizing and protecting cellular structures, including DHN and HSP which will be mentioned below[91].

### Genes annotated as IAA and POD

The expression changes of two IAA genes (Fig. 9D, S3 Table) and two POD genes (Fig. 9E, S3 Table) were partially coincided with the changes of IAA and POD biochemical levels (Table 8). As the biochemical analysis mentioned before, these four genes probably contributed to accelerate bud dormancy release and alleviate injury under cold stress.

### Genes annotated as HSP and histone

The changes between two HSP genes (unigene003256, unigene050870) and two histone genes (unigene025101, unigen029784) expression were inverse during the period from endodormancy to ecodormancy (Fig. 9F and Fig. 9G, S3 Table). The expression of two HSP genes up-regulated before Tre. 2 and after that down-regulated. Dormant buds could synthesize HSPs to protect themselves under cold stress[92], thereby the expression of HSP genes often up-regulate in perennial plants during endodormancy under low temperature. For garlic, HSP genes accounted for the largest proportion of DEGs in vegetative dormant buds and up-regulated during the bud dormancy[21].

Two histone genes, which annotated as HisH2A and HisH3 by NR (S3 Table), down-regulated significantly during the stage of dormancy transition between the time of Tre. 1 and 2, and up-regulated persistently during the stages of ecodormancy and bud elongation (Fig. 9G, S3 Table). The roles of histone genes during the dormancy of seed, bud and tuber have been investigated for many years, but are controversial until now. DNA of eukaryote is packed into chromatin, the basic unit of which is nucleosome, consisting of DNA wound around histone protein complexes[30]. Some important members of histone genes, for instance, Histone H3, are identified as a marker for S-phase progression and supposed to monitor cell cycle progression in the underground dormant buds of perennial leafy spurge[93]. Histone genes are often inhibited under the cold[30], or induced sometimes[94]. A study in Arabidopsis showed that suppression of histone genes expression might contribute to maintain seed dormancy[94]. Another study on leafy spurge revealed that histone genes strongly down-regulate in the late endodormancy or in the early ecodormancy[95]. The patterns of two histone genes expression in our research were consistant with the results of leafy spurge. Unigene025101 and unigen029784 down-regulated from the time of Tre.1 to 2, that were probably induced by the cold in winter, thereby may help to maintain bud dormancy in order to cope with the cold, and play certain roles in bud dormancy transition[93–95].

In addition, histone acetylation is also important for dormancy progression. It is deemed to associate with chromatin remodeling and DNA methylation, both of which are also considered to be involved in endodormancy release[11]. On another hand, some studies confirmed that increase on multi-acetylation of histone genes, such as Histone H3 and H4, might be part of an ordered series of molecular changes that caused the expression of growth-related genes, and ultimately promote to meristem growth. For instance, dormancy release of potato tubers accompanied by histone acetylation[96]. We assumed that up-regulation of our two histone genes in the ecodormant stage were possibly related to acetylation, that were conducive to ecodormancy release and bud growth.

### Genes annotated as LHY

Two genes annotated as LHY were up-regulated before Tre. 5, and down-regulated from then on (Fig. 9H, S3 Table). LHY is one of the key components in circadian clock regulatory pathway[97], which was confirmed to be regulated by light signals in Arabidopsis[98]. LHY genes were repressed in Arabidopsis during the daytime, that induced the genes related to chlorophyll and starch metabolic pathways in allotetraploids or F1 hybrids of Arabidopsis, produced more chlorophyll and starch than their parents in the same environment, and finally led to heterosis in allotetraploids and F1 hybrids which showed growth vigour and increased biomass[97].

Researches are rather limited on the roles and expression of LHY genes in plant dormancy. LHY genes are sensitive to temperature and daylength during poplar dormancy[99]. Down-regulation of LHY genes reduced acclimation of transgenic poplar under freezing temperatures and significantly delayed bud sprouting. For geophytes, temperature and photoperiod strongly affect their dormancy transition, release and bud sprouting, such as garlic[100–104], and also regulate their circadian clock. Transcriptome research on the underground dormant buds of leafy spurge identified another circadian clock gene, namely *CIRCADIAN CLOCK ASSOCIATED 1* (*CCA1*), was up-regulated from paradormancy through to ecodormancy[95].

Based on these useful knowledges, we speculated the homologs of LHY genes in ‘Hangbaishao’ dormant buds were probably bound up with the change of temperature or daylength. Elevation of natural temperature inhibited LHY genes expression, as a result, reduced the cold tolerance of ‘Hangbaishao’ plants when the weather getting warmer after January 21, 2012 (Fig. 2, Fig. 9H, S3 Table). In another aspect, gradual extension of sunshine duration after January 21, 2012 may also induce the down-regulation of LHY genes, thereupon, promote the bud sprouting to a certain degree, that is opposite to the down-regulation of LHY genes caused a delay in bud burst for poplar[99].

### Genes annotated as PHY

PHY genes are typical light-mediated genes, which are senstive to the change of daylength. Just like *LHY* and *CCA1*, PHY genes are also related to the regulation of the circadian clock and endodormancy induction[8,95]. Research on leafy spurge showed that PHY genes were up-regulated from endodormancy to ecodormancy in dormant buds[95,105]. In our study, unigene022263 and unigene010506 down-regulated before Tre. 3, and since then up-regulated obviously (Fig. 9I, S3 Table). The expression patterns of these two PHY genes probably be mediated by the daylength extension, which may contribute to ecodormancy release and bud sprouting.

Based on the results and discussion in each section above mentioned, we can find that many annotated pathways, inner changes on physiology and biochemistry, transcripts of various TFs, highly expressed transcripts and differentially expressed genes, etc., directly or indirectly involved with environmental stress and plant resistance. It is not strange because dormancy itself is an important developmental program for ensuring plants to withstand extended periods of adverse environmental conditions[8,91], such as the cold in winter. The results of DEGs expression are valuable for understanding the molecular mechanism of ‘Hangbaishao’ bud dormancy transition and release, which still need to be validated by other molecular approaches in future research

## Conclusions

*Paeonia lactiflora* ‘Hangbaishao’ is a herbaceous peony cultivar that grows vigorously for many years in Zhejiang Province of China. Under the natural low temperature in Hangzhou, the underground renewal buds of ‘Hangbaishao’ could not accumulate sufficient chilling to break bud dormancy thoroughly until the late January. The transcriptome research on *P*. *lactiflora* ‘Hangbaishao’ described in this paper is the first report on dormancy research based on transcriptome level for herbaceous peonies of the Paeoniaceae family. A total of 207,827 and 51,481 unigenes were obtained with mean lengths of 828.031 and 1250.230 bp using “Trinity” and “Trinity+PRICE”, respectively. The latter strategy was ideal for assembling more integrated unigenes and facilitating subsequent research. Main biochemical pathways, functional classifications, TF families, highly expressed transcripts and SSR motifs were identified and analyzed. The contents of IAA, ABA, and activities of POD and CAT were measured. The expression of 20 interested unigenes annotated as DHN, histone and LHY, etc., were also analyzed. This research was based on multifaceted levels, and the submitted data in NCBI provide valuable resources for understanding the mechanism of bud dormancy transition and release in *P*. *lactiflora*, and also provide a molecular platform for future research on more DEGs expression profiling and network during the dormancy of ‘Hangbaishao’ underground renewal buds.

## Supporting Information

S1 Fig The map of starch and sucrose metabolic pathways. (TIF)

S1 File Amino sequences translated from CDS. (PEP)

S1 Table Pathway annotation of unigenes from ‘Hangbaishao’ transcriptome libraries. (XLSX)

S2 Table KEGG pathway annotation of DEGs during five adjacent dormant phases. (XLSX)

S3 Table Details on expression and annotation of 20 interested unigenes. (XLSX)
