# Supplementary figures and images for: Transcriptomic Analysis of the Underground Renewal Buds during Dormancy Transition and Release in ‘Hangbaishao’ Peony (Paeonia lactiflora)
Source: PLoS One. 2015 Mar 19;10(3):e0119118. doi: 10.1371/journal.pone.0119118 (PMC4366336; doi:10.1371/journal.pone.0119118)

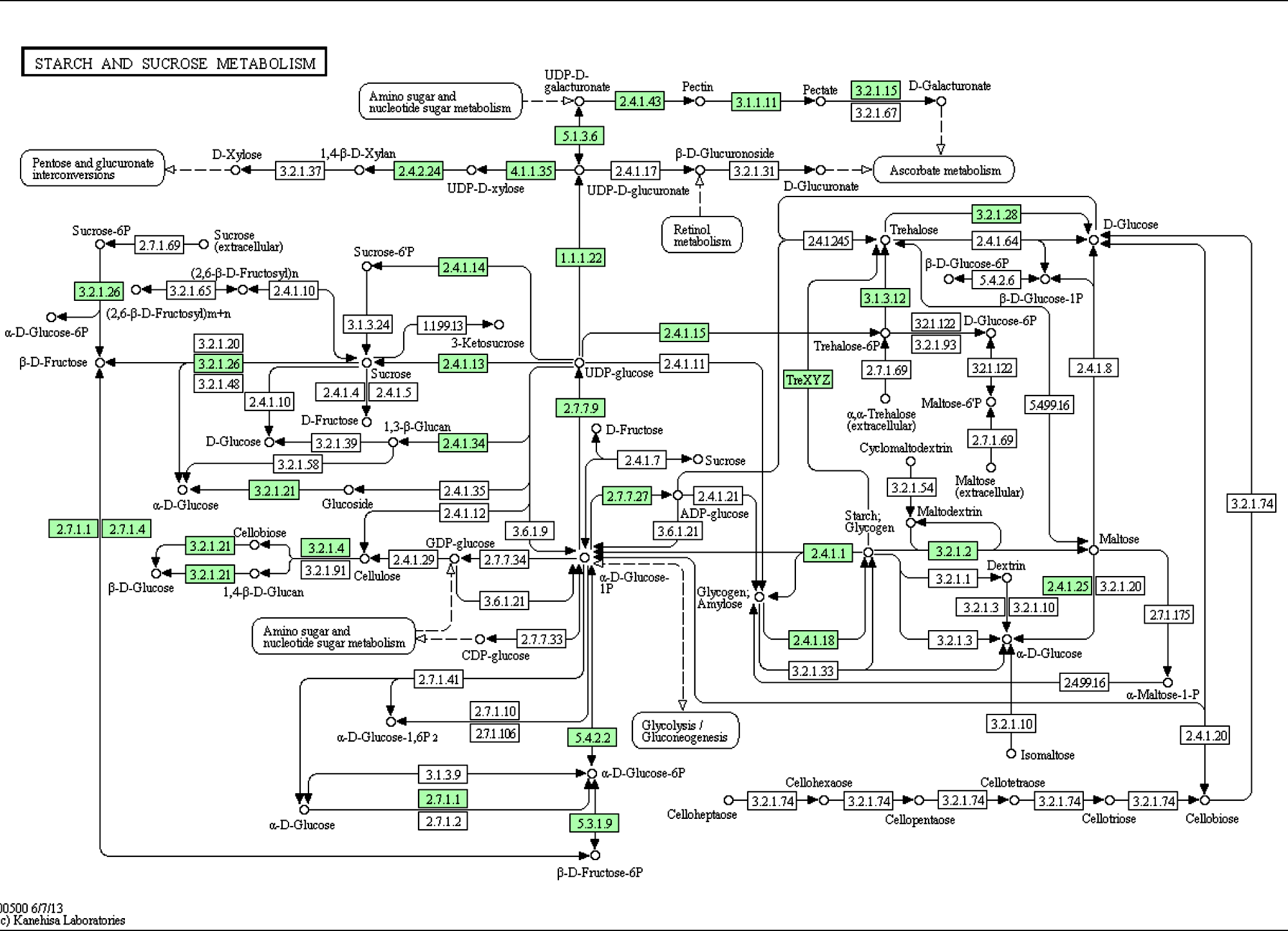

Supplement: S1 Fig — (TIF) [file pone.0119118.s001.tif]
